# Influence of health insurance on withdrawal of life sustaining treatment for patients with isolated traumatic brain injury: a retrospective multi-center observational cohort study

**DOI:** 10.1186/s13054-024-05027-6

**Published:** 2024-07-18

**Authors:** Armaan K. Malhotra, Husain Shakil, Ahmad Essa, Francois Mathieu, Shaurya Taran, Jetan Badhiwala, Yingshi He, Eva Y. Yuan, Abhaya V. Kulkarni, Jefferson R. Wilson, Avery B. Nathens, Christopher D. Witiw

**Affiliations:** 1 Division of Neurosurgery, Unity Health Toronto, St. Michael’s Hospital, 30 Bond Street, Toronto, ON M5B1W8 Canada; 2 Li Ka Shing Knowledge Institute, Toronto, ON Canada; 3 Institute for Health Policy, Management and Evaluation, University of Toronto, Toronto, ON Canada; 4 Division of Orthopedics, Department of Surgery, Shamir Medical Center (Assaf Harofeh), Zerifin, Israel; 5 Faculty of Medicine, Tel Aviv University, Tel Aviv, Israel; 6 Interdepartmental Division of Critical Care Medicine, University of Toronto, Toronto, ON Canada; 7 Division of Neurosurgery, Sunnybrook Health Sciences Centre, Toronto, ON Canada; 8 Division of Neurosurgery, Hospital for Sick Children, Toronto, ON Canada; 9 Division of General Surgery, Sunnybrook Health Sciences Centre, Toronto, ON Canada

**Keywords:** End of life care, Equity, Insurance status, Critical care, Severe traumatic brain injury, Withdrawal of life sustaining treatment

## Abstract

**Background:**

Healthcare inequities for patients with traumatic brain injury (TBI) represent a major priority area for trauma quality improvement. We hypothesized a relationship between health insurance status and timing of withdrawal of life sustaining treatment (WLST) for adults with severe TBI.

**Methods:**

This multicenter retrospective observational cohort study utilized data collected between 2017 and 2020. We identified adult (age ≥ 16) patients with isolated severe TBI admitted participating Trauma Quality Improvement Program centers. We determined the relationship between insurance status (public, private, and uninsured) and the timing of WLST using a competing risk survival analysis framework adjusting for baseline, clinical, injury and trauma center characteristics. Multivariable cause-specific Cox regressions were used to compute adjusted hazard ratios (HR) reflecting timing of WLST, accounting for mortality events. We also quantified the between-center residual variability in WLST using the median odds ratio (MOR) and measured insurance status association with access to rehabilitation at discharge.

**Results:**

We identified 42,111 adults with isolated severe TBI treated across 509 trauma centers across North America. There were 10,771 (25.6%) WLST events in the cohort and a higher unadjusted incidence of WLST events was evident in public insurance patients compared to private or uninsured groups. After adjustment, WLST occurred earlier for publicly insured (HR 1.07, 95% CI 1.02–1.12) and uninsured patients (HR 1.29, 95% CI 1.18–1.41) compared to privately insured patients. Access to rehabilitation was lower for both publicly insured and uninsured patients compared to patients with private insurance. Accounting for case-mix, the MOR was 1.49 (95% CI 1.43–1.55), reflecting significant residual between-center variation in WLST decision-making.

**Conclusions:**

Our findings highlight the presence of disparate WLST practices independently associated with health insurance status. Additionally, these results emphasize between-center variability in WLST, persisting despite adjustments for measurable patient and trauma center characteristics.

**Supplementary Information:**

The online version contains supplementary material available at 10.1186/s13054-024-05027-6.

## Background

The acute phase of TBI is fraught with prognostic uncertainty owing to highly variable and heterogenous outcome trajectories, often ranging from mild residual deficits to death or vegetative states [1, 2]. Clinical decisions made during the acute care phase of TBI patient pathways are critical and carry downstream implications. Withdrawal of life sustaining treatment (WLST) is one such decision that is highly complex; clinicians are often reliant on patient premorbid functional status, injury characteristics and values of family members when navigating care-limiting discussions [3–5]. WLST decision-making is variable at both the provider and facility level [6–8]. An overly nihilistic approach with premature WLST eliminates any prospect of neurological recovery and can introduce self-fulfilling prophecies, while invasive medical treatment in the face of a poor neurological prognosis can prolong suffering, and strain limited healthcare resources [9, 10].

The presence of variable WLST practices reflects the complexity of these decisions for brain injured patients [8]. Socioeconomic disparities in WLST events represent a particularly important domain for focused exploration. Health insurance is a measurable index of socioeconomic status and a potentially modifiable driver of acute care outcomes in a variety of conditions [11–13]. Prior work has highlighted an increased risk for in-hospital mortality among uninsured compared to insured TBI patients, however the influence of health insurance on timing of WLST events remains poorly studied and may represent an important mediator of these discrepancies in observed mortality [14, 15]. We therefore aimed to quantify the association between insurance status and the timing of WLST events in adults with isolated severe TBI. We secondarily sought to assess between-center variability in WLST practices and the association of health insurance with discharge to rehabilitation services and overall mortality. Discovery of meaningful differences in the rate of WLST between insurance groups could provide insight into healthcare disparities and lead to actionable quality improvement targets.

## Methods

### Data source

We utilized data obtained from the American College of Surgeons (ACS) Trauma Quality Improvement Program (TQIP) [16]. This initiative collates trauma-related data from patients presenting to nearly 900 state-designated or ACS verified trauma facilities for the primary goal of enhancing trauma care delivery. TQIP collects data using a standardized methodology for each included subject, including clinical, injury, treatment, and outcome data. Qualified and trained registrars collect the data from the medical records, and quality is assured by data quality reports and internal validation.

### Cohort creation

We identified TQIP eligible adult (age ≥ 16) patients admitted between 2017 and 2020 to participating trauma centers with isolated severe TBI. Notably, patients with a pre-existing advanced directive limiting care are ineligible for inclusion in TQIP. We identified TBI patients using the consensus International Classification for Diseases, tenth revision (ICD-10) codes as recommended by the Center for Disease Control and Prevention (Additional file 4) [17]. Individuals with ICD-10 codes corresponding to TBI were then filtered using the head-region Abbreviated Injury Scale (AIS) score threshold ≥ 3 and Glasgow Coma Scale (GCS) score ≤ 8, which represents a standard technique to classify severe TBI using health administrative data sources [18]. We excluded patients with extracranial injuries AIS ≥ 3 to capture an isolated TBI population and reduce the influence of non-cranial WLST factors. We excluded patients missing primary outcome data (< 1.5% missing WLST) and exposure data (< 2.5% missing primary payment data). We additionally excluded individuals with primary payment method listed as *other* (5.7%), given this was likely a heterogenous group with limited interpretability. Complete case analysis was performed given the proportion of missing data elements for regression variables was low (< 5%) (Additional file 1). We adhered to the REporting of studies Conducted using Observational Routinely collected health Data (RECORD) guidelines [19].

### Exposure

The main study exposure was patient insurance status, which was categorized into private, public, and uninsured groups. Private insurance was defined as private or commercial insurance. Public insurance was determined when the primary payment method was either Medicare, Medicaid, or other governmental insurance.

### Outcomes

The primary study outcome was time to WLST. WLST is reported in TQIP when a decision is made to limit the escalation of active therapy and withdraw ongoing treatments; this includes, but is not limited to, invasive ventilation, hemodialysis, inotrope support or operative interventions. Time to WLST is a separately captured covariate within TQIP. Mortality was defined as a composite measure of in-hospital mortality and hospice transfer. Two secondary outcomes were overall mortality and discharge to a rehabilitation facility. We selected discharge to rehabilitation as a secondary process measure given the importance of rehabilitation access to enhance long-term outcomes for severe TBI patients and prior evidence suggesting variable rehabilitation access in other countries [20–22].

### Covariates

Clinical, injury and hospital covariates were selected based on their clinical relevance. Patient sociodemographic variables were age, sex, and race (black, white, and other). We computed a modified Charlson Comorbidity Index (mCCI) according to prior published adaptations used in TQIP-based studies [23]. Injury details included mechanism of injury, interfacility transfer, GCS total score at emergency department presentation, midline shift on imaging (measured during first 24 h), pupillary responses, receipt of craniotomy or intracranial pressure monitoring (ICP) and year of injury. ICP monitoring and craniotomy were used to understand descriptive unadjusted care intensity across insurance groups, but not included in multivariable modeling given their relationship to WLST as implicit measures of surgeon belief in survival likelihood. Trauma center characteristics were teaching status (non-teaching, academic/university affiliated or community) and profit status (private versus public). Each patient was linked to a deidentified trauma center using unique facility codes.

### Statistical analysis

We performed descriptive comparisons between insurance categories and patient baseline, injury, and center-level characteristics. Standardized differences were used to compare covariates across insurance groups, where a value greater than 0.1 signified a meaningful difference between categories. Data analysis were conducted using R Statistical Software Version 4.2.2 (Vienna, Austria) [24].

To determine the relationship between insurance status and timing of WLST decisions, we applied a competing risk framework with three mutually exclusive outcome states: discharged alive, WLST, and death prior to withdrawal (Additional file 2). A decision for WLST led to death in > 99.5% of cases, meaning survival after withdrawal was not a meaningful state to capture through modeling. Therefore, the main competing risk was mortality without a decision for WLST, which is herein referred to as mortality. Using this framework, we generated risk tables and plotted the non-parametric cause-specific cumulative incidence functions for occurrence of WLST and mortality (competing risk) as functions of time from hospital admission stratified by insurance status. Gray’s test was used to assess for significant differences in the cumulative incidence of WLST and mortality according to insurance status. Surviving patients were censored at discharge from the index TQIP trauma facility.

We constructed multivariable cause-specific Cox models for WLST and mortality. These models control for baseline characteristics, injury factors and trauma center traits to yield cause-specific hazard ratios (HR), which reflect the adjusted rates of WLST, and mortality respectively adjusted for other covariates. These cause-specific models were used to obtain HR estimates for the association between insurance status on the timing of WLST decisions. Robust standard errors were incorporated into multivariable Cox models using unique facility keys to account for inherent differences between treating trauma centers. We visually inspected Schoenfeld residual plots to assess for violations of the proportional hazard assumption.

Secondary objectives were evaluated using multivariable hierarchical logistic regression with outcomes WLST, early WLST (≤ 72 h from admission), overall mortality and discharge to rehabilitation (using the same covariates as cause-specific Cox models). These logistic regression models yield the adjusted odds ratios (OR) for public and uninsured patients compared to patients with private insurance. Inclusion of a random intercept corresponding to unique facility keys allowed us to account for clustering of patients treated within similar centers.

We next estimated the unexplained variation in WLST decision-making across centers. The median odds ratio (MOR) was computed using random intercept variance from the hierarchical logistic model. The MOR can be conceptualized as the odds of a given patient experiencing the outcome of interest when treated at one randomly selected center, compared to another randomly selected center, assuming the patient, injury and center characteristics remain constant [25]. An MOR of one means the likelihood of a particular patient having WLST is the same, irrespective of which hospital they are admitted for management. We plotted the conditional log odds of WLST based on trauma center random intercepts and quantified the number of statistical outliers. Bootstrapping (500 iterations) was performed to estimate 95% confidence intervals for the MOR. An additional random slope model was used to compute the MOR across insurance categories [26].

To explore the association between insurance status and rate of WLST decisions adjusting for the contribution of Medicare beneficiaries, we repeated the primary cause-specific Cox regression analysis using age strata. The cohort was categorized into patients < 65 and ≥ 65 years, in which the latter group represents a higher probability of Medicare coverage.

## Results

There were 42,111 adult patients with isolated severe TBI from 509 trauma centers during the study period (Additional file 1). There were 14,733 (35.0%) patients with private insurance, 20,781 (49.3%) patients with public insurance and 6597 (15.7%) patients that were uninsured (Table 1). The mean cohort age was 49.2 years, and the majority of patients were white males. The mCCI was higher at baseline for public insurance patients compared to private or uninsured insurance groups. Falls (45.6%) and motor vehicle collisions (26.8%) comprised the majority of injury mechanisms. There were 16,863 (40.0%) patients with radiographic midline shift on imaging in the first 24 h after presentation and 2970 (7.1%) had asymmetry in pupillary responses. The proportion of ICP monitors was higher in privately insured patients compared to public or uninsured patients, while the craniotomy rate was lower only among uninsured patients. Within the cohort 10,771 (25.6%) WLST decisions occurred, of which a higher unadjusted proportion took place in public insurance patients compared to private or uninsured groups. There were 18,170 (43.1%) mortalities and 4155 (9.9%) patients discharged to rehabilitation from acute care.

**Table 1:** Study cohort characteristics stratified by insurance status. Maximum standardized differences from pairwise comparisons were summarized

Characteristic	Total (*N* = 42,111)	Private (*N* = 14,733)	Public insurance (*N* = 20,781)	Uninsured (*N* = 6597)	Standardized mean difference
*Baseline and clinical characteristics*					
Male sex	31,001 (73.6%)	11,113 (75.4%)	14,375 (69.2%)	5513 (83.6%)	0.344
Age (Years)					0.987
Mean (SD)	49.2 (21.3)	42.4 (18.6)	57.3 (21.5)	38.7 (15.6)	
Median [Min, Max]	49.0 [16.0, 89.0]	40.0 [16.0, 89.0]	63.0 [16.0, 89.0]	35.0 [16.0, 89.0]	
Race					0.306
Caucasian	29,461 (70.0%)	10,912 (74.1%)	14,600 (70.3%)	3949 (59.9%)	
Black	910 (14.0%)	1558 (10.6%)	2990 (14.4%)	1362 (20.6%)	
Other	6740 (16.0%)	2263 (15.3%)	3191 (15.3%)	1286 (19.5%)	
Modified charlson comorbidity score					0.864
0	19,631 (46.6%)	8484 (57.6%)	6575 (31.6%)	4572 (69.3%)	
1	5139 (12.2%)	2274 (15.4%)	1955 (9.4%)	910 (13.8%)	
2	5911 (14.0%)	2137 (14.5%)	3009 (14.5%)	765 (11.6%)	
> 3	11,430 (27.1%)	1838 (12.5%)	9242 (44.5%)	350 (5.3%)	
Injury characteristics					
Mechanism of injury					0.615
Fall	19,207 (45.6%)	4985 (33.8%)	12,474 (60.0%)	1748 (26.5%)	
Motor vehicle collision	11,272 (26.8%)	5942 (40.3%)	3396 (16.3%)	1934 (29.3%)	
Struck by object	2234 (5.3%)	1090 (7.4%)	811 (3.9%)	333 (5.0%)	
Pedestrian/Cyclist	2708 (6.4%)	823 (5.6%)	1342 (6.5%)	543 (8.2%)	
Firearm	6690 (15.9%)	1893 (12.8%)	2758 (13.3%)	2039 (30.9%)	
Interfacility transfer	14,394 (34.2%)	4306 (29.2%)	8382 (40.3%)	1706 (25.9%)	0.311
Total GCS					0.372
3	26,559 (63.1%)	9442 (64.1%)	12,485 (60.1%)	4632 (70.2%)	
4	2071 (4.9%)	678 (4.6%)	1090 (5.2%)	303 (4.6%)	
5	1735 (4.1%)	582 (4.0%)	917 (4.4%)	236 (3.6%)	
6	4282 (10.2%)	1442 (9.8%)	2361 (11.4%)	479 (7.3%)	
7	4249 (10.1%)	1539 (10.4%)	2177 (10.5%)	533 (8.1%)	
8	3215 (7.6%)	1050 (7.1%)	1751 (8.4%)	414 (6.3%)	
Midline shift					0.672
Yes	16,863 (40.0%)	5058 (34.3%)	9513 (45.8%)	2292 (34.7%)	
No	24,469 (58.1%)	9433 (64.0%)	10,925 (52.6%)	4111 (62.3%)	
Not imaged or missing	779 (1.8%)	242 (1.6%)	343 (1.7%)	194 (2.9%)	
Pupil reactivity					0.789
Both reactive	23,574 (56.0%)	8815 (59.8%)	11,494 (55.3%)	3265 (49.5%)	
One reactive	2970 (7.1%)	1049 (7.1%)	1527 (7.3%)	394 (6.0%)	
Neither reactive	14,101 (33.5%)	4407 (29.9%)	6966 (33.5%)	2728 (41.4%)	
Missing	1466 (3.5%)	462 (3.1%)	794 (3.8%)	210 (3.2%)	
ICP monitoring	9564 (22.7%)	4053 (27.5%)	4228 (20.3%)	1283 (19.4%)	0.191
Craniotomy	6206 (14.7%)	2205 (15.0%)	3242 (15.6%)	759 (11.5%)	0.120
Year of injury					0.045
2017	10,201 (24.2%)	3586 (24.3%)	5006 (24.1%)	1609 (24.4%)	
2018	10,504 (24.9%)	3701 (25.1%)	5109 (24.6%)	1694 (25.7%)	
2019	10,212 (24.3%)	3619 (24.6%)	5057 (24.3%)	1536 (23.3%)	
2020	11,194 (26.6%)	3827 (26.0%)	5609 (27.0%)	1758 (26.6%)	
Center characteristics					
Teaching status					0.071
Academic/University	22,285 (52.9%)	7489 (50.8%)	11,106 (53.4%)	3690 (55.9%)	
Community	14,732 (35.0%)	5306 (36.0%)	7378 (35.5%)	2048 (31.0%)	
Non-teaching	5094 (12.1%)	1938 (13.2%)	2297 (11.1%)	859 (13.0%)	
Hospital profit status					0.183
For-profit	5095 (12.1%)	1742 (11.8%)	2229 (10.7%)	1124 (17.0%)	
Public	37,016 (87.9%)	12,991 (88.2%)	18,552 (89.3%)	5473 (83.0%)	
Outcomes					
Withdrawal of life sustaining treatment	10,771 (25.6%)	2735 (18.6%)	6755 (32.5%)	1281 (19.4%)	0.324
Overall mortality	18,170 (43.1%)	4915 (33.4%)	10,065 (48.4%)	3190 (48.4%)	0.310
Discharge to rehabilitation	4155 (9.9%)	2006 (13.6%)	1760 (8.5%)	389 (5.9%)	0.165

*GCS* Glasgow coma scale; *ICP* Intracranial pressure

### Association between insurance status and WLST

Median time to a WLST decision and mortality were 2 days (interquartile range 1–6 days) and 3 days respectively (interquartile range 2–7 days). Cumulative incidence plots of WLST and mortality are shown in Additional file 3. The unadjusted incidence of WLST events was different across insurance groups (*p* < 0.001). At day 2 after injury, 17% of public insurance patients, 10% of uninsured patients and 8.5% of private insurance patients had WLST; by day 5 these proportions were 24, 15 and 13% respectively (Additional file 5). The unadjusted mortality incidence was also different between insurance groups (*p* < 0.001) and highest among uninsured patients over time. Over the first 10 days after injury, the unadjusted mortality incidence among uninsured patients was over two-fold the incidence observed in public and private insurance patients (Additional file 5).

**Fig. 1:**
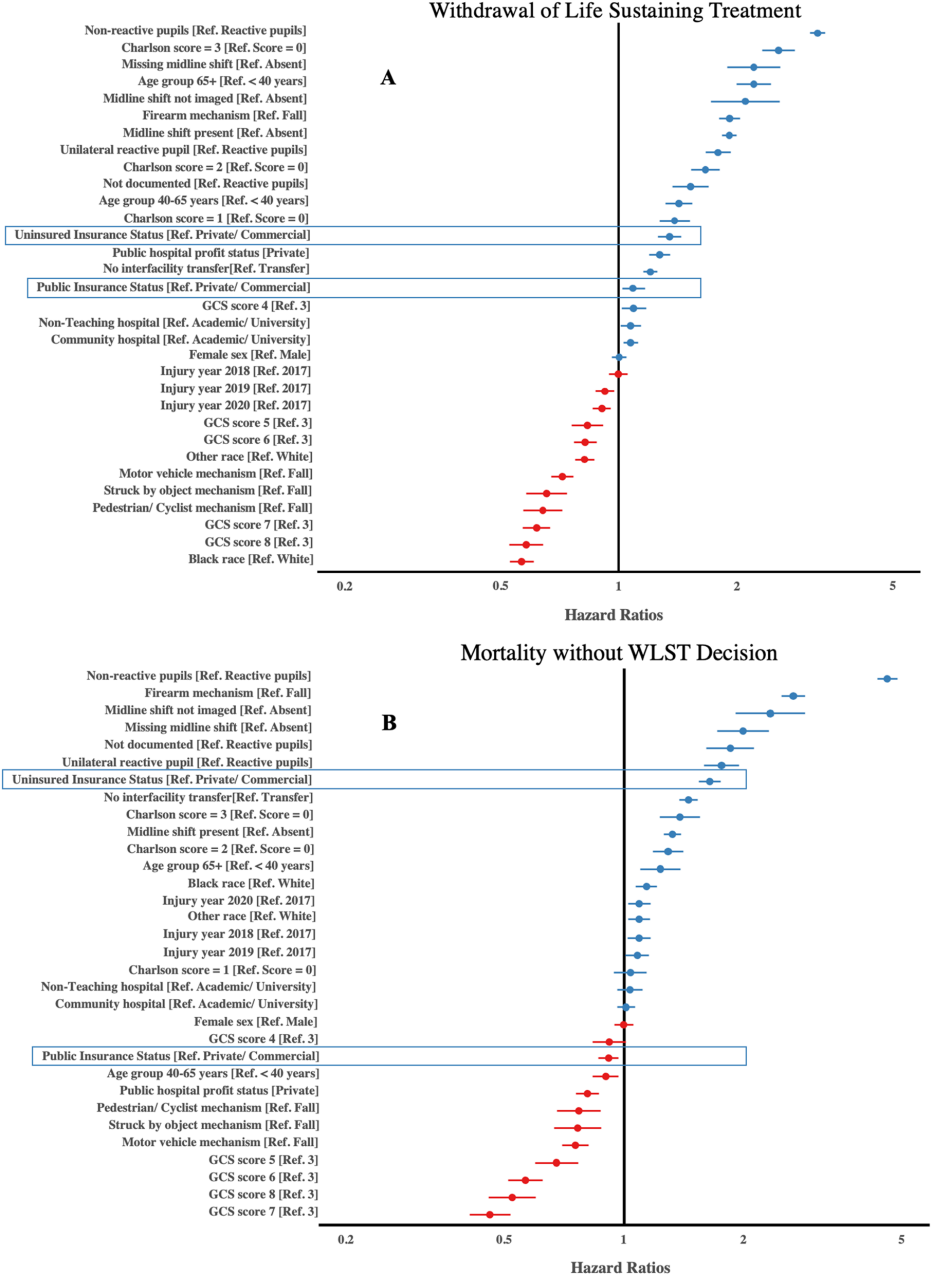
Forest plot depicting multivariable cause-specific Cox model. The model estimates (cause-specific hazard ratios, HR) for withdrawal of life sustaining treatment (WLST) and mortality without decision for WLST (panels A and B respectively). *GCS*, Glasgow Coma Scale

After adjustment for baseline, clinical, injury and hospital characteristics, the cause-specific HR for WLST was higher in publicly insured patients (HR 1.07, 95% CI 1.02–1.12) and uninsured patients (HR 1.29, 95% CI 1.18–1.41) compared to patients with private insurance (Table 2). Stated another way, there was an independently shorter time to WLST decision in publicly insured and uninsured patient populations. Adjusted HRs for remaining covariates are summarized in Fig. 1 and Additional file 6. Hierarchical logistic regression also demonstrated increased adjusted odds for WLST among uninsured patients and patients with public insurance compared to patients with private insurance (Table 3).

**Table 2:** Summary of multivariable cause-specific Cox model main results

Insurance status	Cause-specific Hazard ratio	95% CI
*WLST*		
Private	–	–
Public	1.07	1.02–1.12
Uninsured	1.29	1.18–1.41
*Mortality without decision for WLST*		
Private	–	–
Public	0.92	0.86–0.98
Uninsured	1.66	1.54–1.79

Regression results shown and cause-specific hazard ratios for withdrawal of life sustaining treatment (WLST) and mortality without WLST respectively (competing risk)

*WLST* Withdrawal of life sustaining treatment; *CI* Confidence interval

**Table 3:** Summary from multivariable hierarchical logistic regression sensitivity analyses

Insurance status	Odds ratio	95% CI
*WLST*		
Private	**–**	**–**
Public	1.11	1.04–1.18
Uninsured	1.12	1.03–1.23
*Early WLST (≤ 72 h)*		
Private	**–**	**–**
Public	1.08	1.00–1.18
Uninsured	1.23	1.11–1.37
*Overall mortality*		
Private	**–**	**–**
Public	1.03	0.97–1.10
Uninsured	1.81	1.66–1.96
*Discharge to rehabilitation*		
Private	**–**	**–**
Public	0.82	0.76–0.90
Uninsured	0.39	0.34–0.44

Assessing adjusted odds of withdrawal of life sustaining treatment (WLST), overall mortality, and discharge to rehabilitation services across insurance levels

*WLST* Withdrawal of life sustaining treatment; *CI* Confidence interval

### Secondary outcomes

The adjusted mortality rate was lower among publicly insured patients and higher among uninsured patients compared to private insurance patients (Table 2). Hierarchical logistic regression models were constructed to determine the adjusted odds of early WLST (≤ 72 h), overall mortality and discharge to rehabilitation across insurance groups. Death and early WLST were more likely in uninsured patients compared to patients with private insurance; no difference was demonstrated between public and private insurance groups for death, but early WLST was more likely in public compared to private insurance patients. The adjusted odds of discharge to a rehabilitation facility were significantly lower for both public insurance and uninsured patients compared to private insurance patients (Table 3).

### Inter-hospital variability

**Fig. 2:**
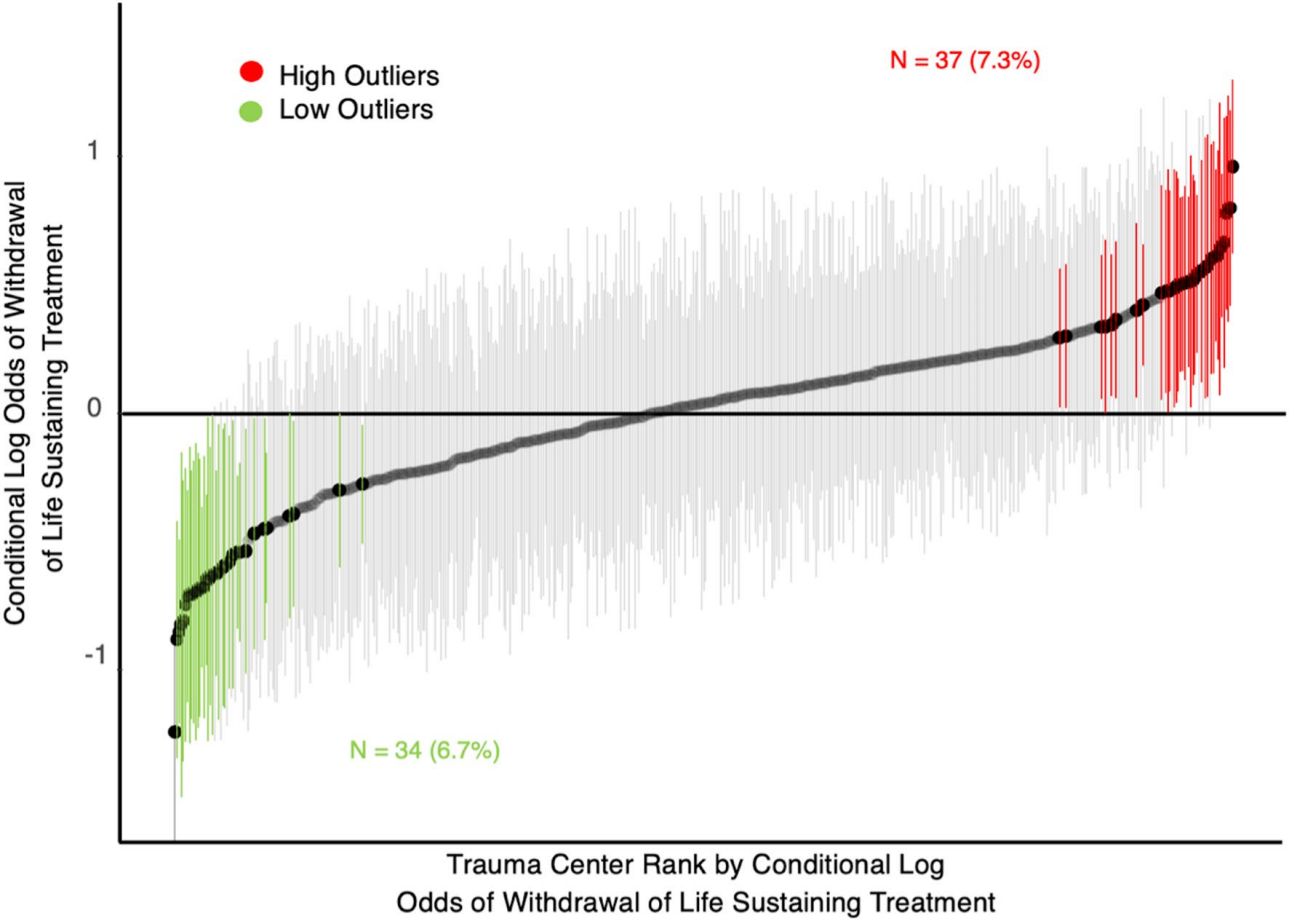
Trauma center variability associated with withdrawal of life sustaining treatment for severe traumatic brain injury. Trauma hospitals ranked by conditional log odds of withdrawal of life sustaining treatment controlling for patient, injury, and hospital-level characteristics from hierarchical logistic regression model. Centers in red are high outlier centers and those in green are low outlier centers

After accounting for patient and hospital fixed effects (sociodemographic, clinical, injury and trauma center variables), the MOR was 1.49 (95% CI 1.43–1.55). This means that on average, an adult with isolated severe TBI managed in two randomly selected trauma centers with high versus low tendency for WLST has an approximately 50% increased chance of undergoing withdrawal based on trauma center variability alone. Despite adjustment for patient and trauma center characteristics, there remains unexplained between-center variability in WLST decisions. This relationship was plotted in Fig. 2, in which 71 trauma centers (13.9% of included centers) were either high or low outliers with respect to their tendency to perform WLST. The MOR for private insurance, public insurance, and uninsured groups were 1.33, 1.50 and 1.65 respectively.

### Sensitivity analysis

Age-stratified multivariable Cox regression results are summarized in Additional file 7. After adjustment, uninsured patients experienced significantly shorter time to both decision for WLST and mortality compared to privately insured patients in both age strata. Among patients < 65 years, there was no difference between the rate of WLST decisions across public and private insurance categories; the rate of mortalities was lower in public compared private insurance patients. WLST decisions occurred significantly faster in Medicare beneficiaries compared to privately insured older patients with no difference in mortality rates.

## Discussion

Accounting for competing risks, uninsured patients experience earlier WLST compared to privately insured counterparts after adjustment for patient, injury, and hospital characteristics. The adjusted rate of WLST is also higher in publicly insured patients compared to privately insured patients; this effect is largely driven by differences among older adults covered by Medicare. We estimated an MOR of 1.49, suggesting substantial between-center variability in WLST decision-making; this variability was largest in magnitude for uninsured patients. We additionally highlight the presence of disparate access to rehabilitation services at discharge for publicly insured and uninsured patients compared to their privately insured counterparts.

Prior groups have highlighted that uninsured patients with moderate and severe TBI experience higher in-hospital mortality, but did not explore WLST decision-making differences as a potential mediator of this observed effect [14, 15, 27]. The results of our study remain consistent with prior work using TQIP data assessing factors associated with WLST, however provide a more robust analysis accounting for the competing risk of mortality in the absence of a decision for WLST [3, 28]. Our work additionally emphasizes that withdrawal decisions occur earlier during hospitalization for publicly insured and uninsured patient groups compared to privately insured groups. Another cross-sectional study of TBI survivors treated within the same trauma center highlighted that patients with government insurance experienced higher levels of disability one year after injury compared to patients with private insurance [29]. Their work suggests that care delivery differences may persist after discharge from acute care settings in the form of differential rehabilitation service access. Our secondary analysis underscores this finding and demonstrates differential inpatient rehabilitation access according to health insurance status.

The median time to WLST decision was 2 days, which is earlier than the recommended 72-h threshold suggested by guidelines and observational evidence for neuro-prognostication [30–32]. Additionally, differences in the time of WLST decision-making have important implications, and might result in self-fulfilling prophecies. The concept of clinical nihilism in neurological emergencies is not new; it represents a decision-making framework motivated by low confidence in the ability for medical or surgical interventions to meaningfully change an anticipated poor outcome [33, 34]. While the potential for unmeasured confounding exists, our findings illustrate that differences in insurance status are independently associated with shorter timing of mortality events associated with WLST decisions. In a recent survey conducted at the Seattle International Severe Traumatic Brain Injury Consensus Conference of neurotrauma experts, over 60 percent of respondents stated WLST was justifiable among patients who would go on to recover to a lower-severe disability state as measured on the Glasgow Outcome Scale Extended scale [10]. This relative equipoise among a highly specialized group of providers highlights a major challenge in promoting equal access to the prospect of neuro-recovery among TBI patients [35]. The same study highlighted 66% of respondents felt very concerned about therapeutic nihilism related to severe TBI patient management in centers around the world [10].

Similarly, surveys of TBI survivors with severe disability demonstrate roughly half of patients report that their health-related quality of life falls within normative ranges, emphasizing the disability paradox between acute care decision-makers and long-term patient reported outcomes [36]. There is an urgent need for focused qualitative research to explore patient, provider and family beliefs about end of life decision making and future disability to inform practice recommendations.

There were several limitations with this work. We encountered missing data during cohort creation, which introduces the potential for biased effect estimates. This is mitigated in part by the rigorous data quality standards employed by ACS TQIP and overall low proportion of missing data elements (< 5%). Notably, the proportion of missing primary outcome and exposure data were also very low, suggesting the influence of this missing data on our inferences was minor, if present. Due to the observational nature of this study, there remains the potential for unmeasured confounding, particularly related to social marginalization and poverty, which may influence the exposure-outcome relationship (especially among uninsured patients). Despite this, we were able to adjust for a number of meaningful clinical, injury and trauma center confounders; additional logistic regression analyses added robustness to main inferences by supporting the primary analysis. The ACS/state verification process for trauma centers implies adherence to high quality standards and evidence-based practice guidelines to enhance care delivery; the disparities in WLST decision-making are likely underestimated for non-ACS TQIP facilities.

## Conclusions

This work highlights the presence of healthcare inequities in trauma provision for adults with severe TBI. We demonstrate insurance status is associated with differences in timing of WLST, access to rehabilitation and overall mortality. Our findings recapitulate the existence of between-center differences in the propensity for WLST decision-making, suggesting the urgent need for qualitative research to explore this variability at the trauma center and provider levels.

### Supplementary Information

Additional file 1.

Additional file 2.

Additional file 3.

Additional file 4.

Additional file 5.

Additional file 6.

Additional file 7.

## Data Availability

The data described through this study were made available following request from the American College of Surgeons (ACS) Trauma Quality Improvement Program (TQIP). There are notable restrictions that apply to data availability, which were utilized with specific approval for the presented work and are not publicly accessible. Qualified investigators may place requests for the data, which may be available with permission of the ACS.

## Abbreviations

ACS: American college of surgeons
AIS: Abbreviated injury scale
GCS: Glasgow coma scale
HR: Hazard ratios
ICD-10: The consensus international classification for diseases, tenth revision
ICP: Intracranial pressure
MOR: Median odds ratio
mCCI: Modified Charlson comorbidity index
OR: Odds ratios
RECORD: The REporting of studies Conducted using Observational Routinely collected health Data
TBI: Traumatic brain injury
TQIP: Trauma quality improvement program
WLST: Withdrawal of life sustaining treatment
