# Supplementary material for: Influence of health insurance on withdrawal of life sustaining treatment for patients with isolated traumatic brain injury: a retrospective multi-center observational cohort study
Source: Crit Care. 2024 Jul 18;28:251. doi: 10.1186/s13054-024-05027-6 (PMC11264615; doi:10.1186/s13054-024-05027-6)
Supplement: Supplementary file 1 — Additional file 1. [file 13054_2024_5027_MOESM1_ESM.pdf]

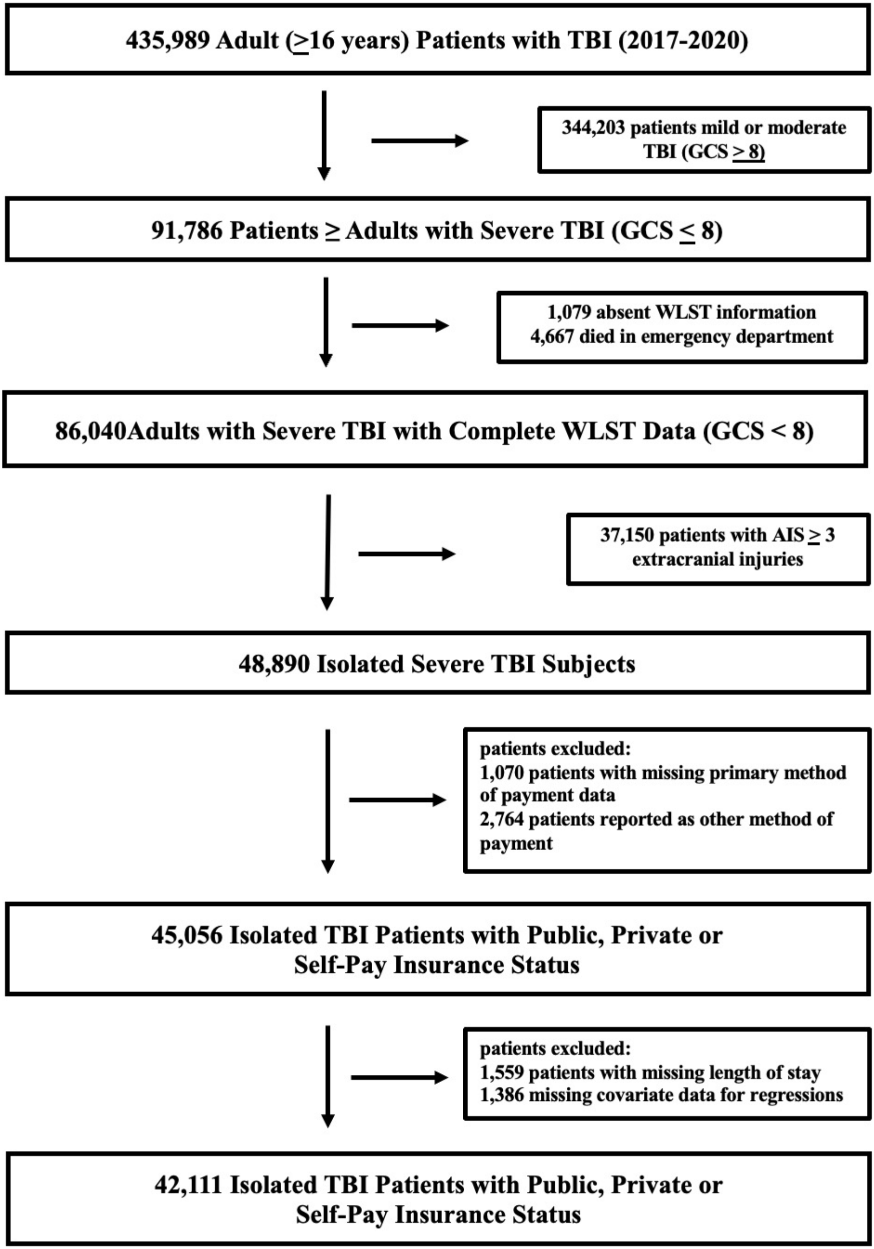
435,989 Adult (≥16 years) Patients with TBI (2017-2020)
344,203 patients mild or moderate TBI (GCS ≥ 8)
91,786 Patients ≥ Adults with Severe TBI (GCS ≤ 8)
1,079 absent WLST information
4,667 died in emergency department
86,040Adults with Severe TBI with Complete WLST Data (GCS < 8)
37,150 patients with AIS ≥ 3 extracranial injuries
48,890 Isolated Severe TBI Subjects
patients excluded:
1,070 patients with missing primary method of payment data
2,764 patients reported as other method of payment
45,056 Isolated TBI Patients with Public, Private or Self-Pay Insurance Status
patients excluded:
1,559 patients with missing length of stay
1,386 missing covariate data for regressions
42,111 Isolated TBI Patients with Public, Private or Self-Pay Insurance Status
