# Supplementary figures and images for: Influence of health insurance on withdrawal of life sustaining treatment for patients with isolated traumatic brain injury: a retrospective multi-center observational cohort study
Source: Crit Care. 2024 Jul 18;28:251. doi: 10.1186/s13054-024-05027-6 (PMC11264615; doi:10.1186/s13054-024-05027-6)

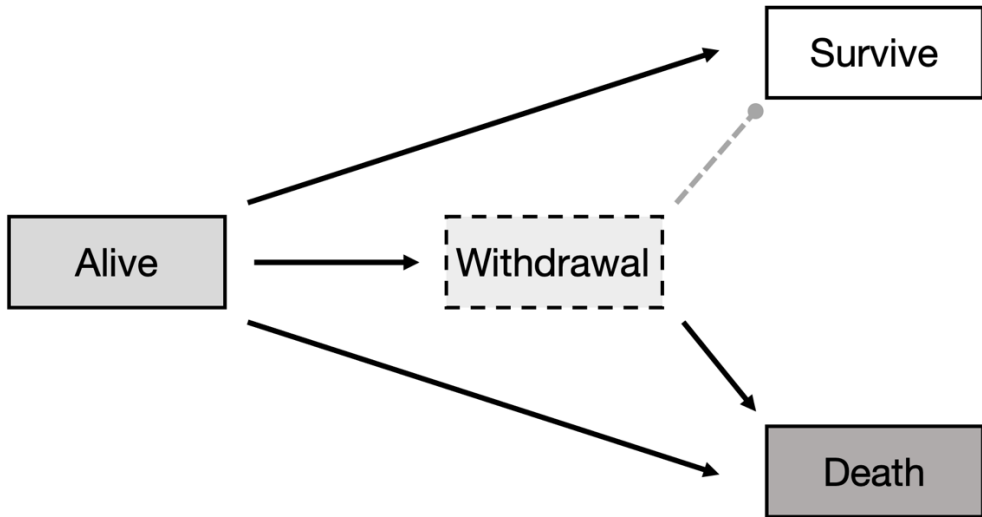

Supplement: Supplementary file 2 — Additional file 2. [file 13054_2024_5027_MOESM2_ESM.pdf]

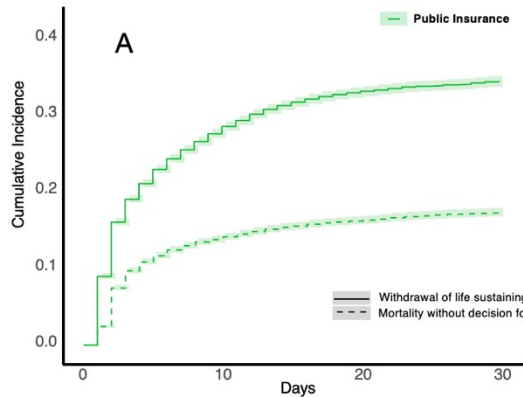

| At Risk |       |      |      |       |
|---------|-------|------|------|-------|
| Public  | 20781 | 8546 | 4043 | 1990  |
| Public  | 0     | 9000 | 9878 | 10035 |

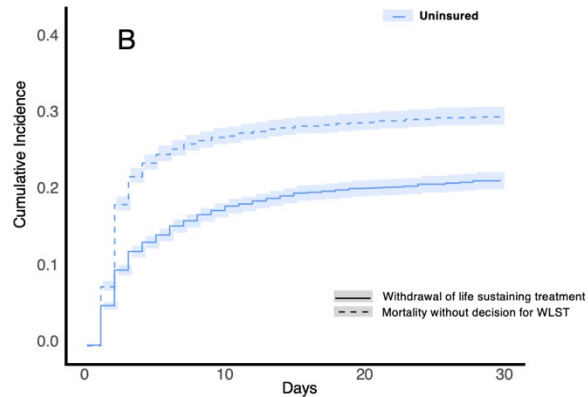

| At Risk   |      |      |      |      |
|-----------|------|------|------|------|
| Uninsured | 6597 | 2125 | 1117 | 634  |
| Uninsured | 0    | 2998 | 3142 | 3175 |

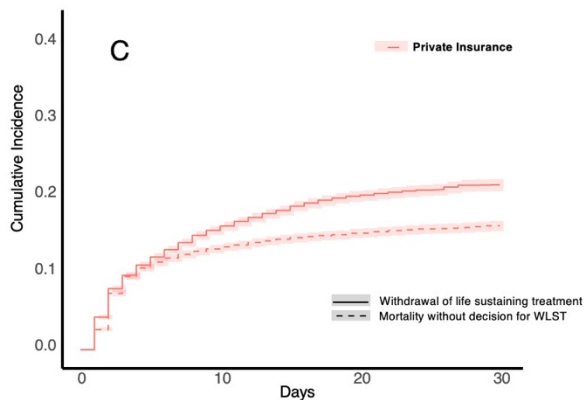

| At Risk |       |      |      |      |
|---------|-------|------|------|------|
| Private | 14733 | 6941 | 3258 | 1417 |
| Private | 0     | 4323 | 4796 | 4890 |

Supplement: Supplementary file 3 — Additional file 3. [file 13054_2024_5027_MOESM3_ESM.pdf]
