# Supplementary material for: Influence of health insurance on withdrawal of life sustaining treatment for patients with isolated traumatic brain injury: a retrospective multi-center observational cohort study
Source: Crit Care. 2024 Jul 18;28:251. doi: 10.1186/s13054-024-05027-6 (PMC11264615; doi:10.1186/s13054-024-05027-6)
Supplement: Supplementary file 4 — Additional file 4. [file 13054_2024_5027_MOESM4_ESM.docx]

**Additional File 4. International Classification of Disease codes (version 10) corresponding to traumatic brain injury.**

| **ICD-10 Code** | **Description** |
| --- | --- |
| S02.0, S02.1- | Fracture of skull |
| S04.02, S04.03-, S04.04- | Injury to optic chiasm, optic tract, optic pathways, or visual cortex |
| S06- | Intracranial injury |
| S07.1 | Crushing injury of the skull |
| S02.8, S02.9- | Other specified fracture of skull or facial bones |
| Excluded T74.4 | Shaken baby syndrome |

Abbreviations: ICD-10, International Classification for Diseases, tenth revision.
