# Supplementary material for: Influence of health insurance on withdrawal of life sustaining treatment for patients with isolated traumatic brain injury: a retrospective multi-center observational cohort study
Source: Crit Care. 2024 Jul 18;28:251. doi: 10.1186/s13054-024-05027-6 (PMC11264615; doi:10.1186/s13054-024-05027-6)
Supplement: Supplementary file 5 — Additional file 5. [file 13054_2024_5027_MOESM5_ESM.docx]

**Additional File 5. Cumulative incidence table highlighting differences between overall event incidence across withdrawal of life sustaining treatment (WLST) and mortality without WLST (competing risk) groups stratified by insurance status.**

| **Time** | **Day 2** | **Day 5** | **Day 10** | **Day 20** | **Day 30** | **p-value*^1^*** |
| --- | --- | --- | --- | --- | --- | --- |
| **WLST Decision** | | | | | | |
| **Insurance Status** |  |  |  |  |  | <0.001 |
| Private Insurance | 8.5% (8.0%, 8.9%) | 13% (12%, 13%) | 17% (17%, 18%) | 21% (21%, 22%) | 23% (22%, 24%) |  |
| Public Insurance | 17% (17%, 18%) | 24% (24%, 25%) | 30% (30%, 31%) | 35% (35%, 36%) | 37% (36%, 37%) |  |
| Uninsured | 10% (9.7%, 11%) | 15% (14%, 16%) | 19% (18%, 20%) | 22% (21%, 23%) | 23% (22%, 24%) |  |
| **Mortality in Absence of WLST Decision** | | | | | | |
| **Insurance Status** |  |  |  |  |  | <0.001 |
| Private Insurance | 7.8% (7.4%, 8.2%) | 12% (12%, 13%) | 14% (14%, 15%) | 16% (15%, 17%) | 17% (16%, 18%) |  |
| Public Insurance | 7.9% (7.6%, 8.3%) | 12% (12%, 13%) | 15% (15%, 16%) | 17% (17%, 18%) | 18% (18%, 19%) |  |
| Uninsured | 20% (19%, 21%) | 26% (25%, 28%) | 29% (28%, 30%) | 31% (30%, 32%) | 32% (30%, 33%) |  |

P values reflect Gray’s test result comparing cause-specific cumulative incidence functions.

Values in parentheses reflect 95% confidence intervals.

Abbreviations: WLST, withdraw life sustaining treatment.
