# Supplementary material for: Influence of health insurance on withdrawal of life sustaining treatment for patients with isolated traumatic brain injury: a retrospective multi-center observational cohort study
Source: Crit Care. 2024 Jul 18;28:251. doi: 10.1186/s13054-024-05027-6 (PMC11264615; doi:10.1186/s13054-024-05027-6)
Supplement: Supplementary file 6 — Additional file 6. [file 13054_2024_5027_MOESM6_ESM.docx]

**Additional File 6. Multivariable cause-specific Cox model results.**

| **Characteristic** | **WLST** | | **Mortality without WLST** | |
| --- | --- | --- | --- | --- |
|  | **Cause-Specific HR** | **95% CI** | **Cause-Specific HR** | **95% CI** |
| Age Category (ref. < 40 years) |  |  |  |  |
| 40 to 65 years | 1.45 | 1.34, 1.56 | 0.91 | 0.85, 0.98 |
| > 65 years | 2.24 | 2.03, 2.47 | 1.25 | 1.11, 1.42 |
| Female Sex | 1.01 | 0.96, 1.05 | 1.00 | 0.95, 1.06 |
| Race (ref. White) |  |  |  |  |
| Black | 0.57 | 0.51, 0.62 | 1.11 | 1.04, 1.20 |
| Other | 0.82 | 0.76, 0.88 | 1.08 | 1.01, 1.16 |
| Insurance (ref. Private) |  |  |  |  |
| Public | 1.07 | 1.02, 1.12 | 0.92 | 0.86, 0.98 |
| Uninsured | 1.29 | 1.18, 1.41 | 1.66 | 1.54, 1.79 |
| Mechanism of Injury (ref. Fall) |  |  |  |  |
| Firearm | 1.89 | 1.75, 2.03 | 2.70 | 2.51, 2.91 |
| Motor vehicle | 0.72 | 0.68, 0.77 | 0.75 | 0.69, 0.82 |
| Pedestrian/ cyclist | 0.64 | 0.57, 0.72 | 0.76 | 0.67, 0.86 |
| Struck object | 0.65 | 0.57, 0.73 | 0.76 | 0.65, 0.87 |
| Charlson Comorbidity Index Score (ref. 0) |  |  |  |  |
| 1 | 1.37 | 1.26, 1.50 | 1.04 | 0.93, 1.16 |
| 2 | 1.63 | 1.50, 1.77 | 1.30 | 1.18, 1.43 |
| 3 | 2.51 | 2.29, 2.75 | 1.43 | 1.27, 1.62 |
| Hospital Teaching Status (ref. academic/ university) |  |  |  |  |
| Community | 1.07 | 0.98, 1.16 | 1.01 | 0.92, 1.10 |
| Non-Teaching | 1.06 | 0.95, 1.18 | 1.04 | 0.93, 1.16 |
| Hospital Payment Type (ref. For Profit) |  |  |  |  |
| Public | 1.29 | 1.14, 1.46 | 0.82 | 0.74, 0.92 |
| GCS Score (ref. GCS=3) |  |  |  |  |
| 4 | 1.09 | 1.01, 1.19 | 0.92 | 0.83, 1.02 |
| 5 | 0.86 | 0.78, 0.95 | 0.68 | 0.59, 0.77 |
| 6 | 0.85 | 0.79, 0.91 | 0.56 | 0.51, 0.62 |
| 7 | 0.63 | 0.58, 0.68 | 0.45 | 0.40, 0.52 |
| 8 | 0.60 | 0.54, 0.66 | 0.52 | 0.45, 0.60 |
| No Interfacility Transfer | 1.18 | 1.12, 1.25 | 1.45 | 1.37, 1.55 |
| Injury Year (ref. 2017) |  |  |  |  |
| 2018 | 1.00 | 0.94, 1.06 | 1.09 | 1.01, 1.17 |
| 2019 | 0.92 | 0.86, 0.98 | 1.08 | 1.00, 1.17 |
| 2020 | 0.90 | 0.84, 0.97 | 1.11 | 1.03, 1.20 |
| Pupillary Responses (ref. Both reactive) |  |  |  |  |
| Not Documented | 1.51 | 1.33, 1.71 | 1.84 | 1.57, 2.17 |
| Neither reactive | 3.17 | 2.99, 3.35 | 4.75 | 4.44, 5.09 |
| One reactive | 1.80 | 1.66, 1.96 | 1.78 | 1.59, 1.99 |
| Midline Shift (ref. Absent) |  |  |  |  |
| Missing | 2.23 | 1.87, 2.66 | 2.05 | 1.70, 2.46 |
| Not Imaged | 1.97 | 1.59, 2.43 | 2.32 | 1.96, 2.93 |
| Present | 1.90 | 1.81, 2.00 | 1.35 | 1.28, 1.43 |

Fixed effects estimates (cause-specific hazard ratios, HR) for withdrawal of life sustaining treatment (WLST) and mortality without decision for WLST (competing risk) are respectively summarized.

Abbreviations: WLST, withdraw life sustaining treatment; HR, hazard ratio; CI, confidence interval; GCS, Glasgow Coma Scale.
