# Supplementary material for: Influence of health insurance on withdrawal of life sustaining treatment for patients with isolated traumatic brain injury: a retrospective multi-center observational cohort study
Source: Crit Care. 2024 Jul 18;28:251. doi: 10.1186/s13054-024-05027-6 (PMC11264615; doi:10.1186/s13054-024-05027-6)
Supplement: Supplementary file 7 — Additional file 7. [file 13054_2024_5027_MOESM7_ESM.docx]

**Additional File 7. Multivariable cause-specific Cox model results for age strata.**

| **Age < 65 years** | | |
| --- | --- | --- |
| ***Insurance Status*** | ***Cause-Specific Hazard Ratio*** | ***95% CI*** |
| **Withdrawal of Life Sustaining Therapy** | |  |
| Private | - | - |
| Public | 1.00 | 0.93-1.07 |
| Uninsured | **1.24** | **1.13-1.36** |
| **Mortality without WLST** | | |
| Private | - | - |
| Public | **0.89** | **0.83-0.95** |
| Uninsured | **1.58** | **1.47-1.70** |
| **Age > 65 years** | | |
| **Withdrawal of Life Sustaining Therapy** | | |
| Private | - | - |
| Public | **1.16** | **1.06-1.28** |
| Uninsured | **1.32** | **1.08-1.62** |
| **Mortality without WLST** | | |
| Private | - | - |
| Public | 1.05 | 0.93-1.19 |
| Uninsured | **1.84** | **1.42-2.38** |

Regression results shown for withdrawal of life sustaining treatment (WLST) and mortality without decision for WLST respectively (competing risk).

Abbreviations: WLST, withdraw life sustaining treatment; CI, confidence interval.
